# Quantifying the Potential for Future Gene Therapy to Lower Lifetime Risk of Polygenic Late-Onset Diseases

**DOI:** 10.3390/ijms20133352

**Published:** 2019-07-08

**Authors:** Roman Teo Oliynyk

**Affiliations:** 1 Centre for Computational Evolution, University of Auckland, Auckland 1010, New Zealand; roli573@aucklanduni.ac.nz; 2 Department of Computer Science, University of Auckland, Auckland 1010, New Zealand

**Keywords:** polygenic risk, heritability, late-onset disease, simulation, gene therapy, gene editing, lifetime risk, life expectancy

## Abstract

Gene therapy techniques and genetic knowledge may sufficiently advance, within the next few decades, to support prophylactic gene therapy for the prevention of polygenic late-onset diseases. The risk of these diseases may, hypothetically, be lowered by correcting the effects of a subset of common low effect gene variants. In this paper, simulations show that if such gene therapy were to become technically possible; and if the incidences of the treated diseases follow the proportional hazards model with a multiplicative genetic architecture composed of a sufficient number of common small effect gene variants, then: (a) late-onset diseases with the highest familial heritability will have the largest number of variants available for editing; (b) diseases that currently have the highest lifetime risk, particularly those with the highest incidence rate continuing into older ages, will prove the most challenging cases in lowering lifetime risk and delaying the age of onset at a population-wide level; (c) diseases that are characterized by the lowest lifetime risk will show the strongest and longest-lasting response to such therapies; and (d) longer life expectancy is associated with a higher lifetime risk of these diseases, and this tendency, while delayed, will continue after therapy.

## 1. Introduction

In the past two decades, the human genome has been successfully sequenced. Whole genome sequencing (WGS) and genome-wide association studies (GWASs) of human genomes (as well as those of other organisms) have become an everyday occurrence [1]. Our knowledge of genetic variants, particularly the single nucleotide polymorphisms (SNPs) associated with susceptibility to diseases, has become deeper and more extensive.

Experimental gene therapy techniques, aimed at diseases caused by a single defective gene or a single SNP—the so-called Mendelian conditions—are being refined. Mendelian conditions cause high mortality and morbidity, but each of these conditions affects only a minute fraction of the population. As of June 2019, the OMIM Gene Map Statistics [2] compendium has listed 6436 phenotypic genetic conditions caused by 4102 gene mutations. This list includes a variety of conditions, with onsets ranging from very early to late. For example, type 1 diabetes mellitus is caused by single defects in the HLA-DQA1, HLA-DQB1, or HLA-DRB1 genes [3]. Early-onset Alzheimer’s disease is caused primarily by APP, PSEN1, or PSEN2 gene mutations and affects a relatively small proportion of the population, starting in their thirties, with the majority of mutation carriers being affected by the age of 65 [4]. In contrast, macular degeneration [5,6,7] is primarily caused by a small number of high-effect variants and manifests at a relatively old age. In some cases, individualized genetic diagnoses, where an SNP that needs to be edited can be specified precisely, are possible. Over the last two decades, 287 monogenic disease clinical trials have been conducted worldwide [8]. When the medical technology becomes available, individuals who receive treatment will be effectively cured and will have no need for concern about the single specific cause of their disease.

Polygenic or complex late-onset diseases (LODs) pose a more nuanced problem, and this study will focus on them. There are thousands of estimated gene variants or SNPs of typically small effect that, in combination, constitute the polygenic LOD risk of each individual [9,10]. These diseases include the old-age diseases that eventually affect most individuals and are exemplified by cardiovascular disease (particularly coronary artery disease (CAD)), cerebral stroke, type 2 diabetes (T2D), senile dementia, Alzheimer’s disease (AD), cancer, and osteoarthritis.

What distinguishes polygenic LODs from infectious diseases or from Mendelian genetic conditions is difficulty in terms of the concept of cure. The diseases of aging are primarily a consequence of an organism’s decline over time, leading to increased susceptibility to many LODs [11,12,13]. The combination of genetic liability, environmental factors, and the physiological decline of multiple organ systems leads to individual disease presentations [14]. Detrimental gene variants are exacerbating factors [15], compared to the average distribution of common gene variants that define human conditions, as they apply to polygenic LODs. The time of onset for each individual is modulated by genotype and environment [16]. While some individuals will be diagnosed at a relatively young age, others will not be diagnosed with a particular LOD during their lifetime [17]. According to the current consensus, a large number of common low-effect variants offer the likeliest explanation for the heritability of the majority of complex traits [18,19]. For example, in the cancers analyzed in this study, the fraction of all diagnoses that were attributed to highly detrimental inherited mutations was relatively low—it was estimated to explain heritability connected with 10%–14% of breast cancers [20,21], 10%–12% of prostate cancers [22,23,24,25], 5%–10% of colorectal cancers [26,27], and was assumed to be a relatively minor fraction for lung cancers [28,29,30]. For the majority of these cancers, liability is attributed to the common low-effect gene variants and environmental factors. The development of cancer is a multistage process, wherein individual variability in any tumorigenesis stage duration or liability may be influenced by hereditary predisposition, as well as environmental factors [31]. The level of susceptibility to the major polygenic LODs, and the difference between high-risk and low-risk individuals, may lie in a slightly higher- or lower-than-average fraction of detrimental gene variants. Certainly, the failure does not begin immediately prior to the age of diagnosis. For example, AD deterioration begins decades before symptoms first become noticeable [32]. A similar situation holds for cardiovascular disease [33,34] and cancer [35].

The best cure is prevention, and the time may be nearing when prophylactic gene therapy will be attempted for the prevention of complex polygenic diseases. Much scientific knowledge and technical expertise is required and many ethics questions will need to be settled before this level of prophylactic gene therapy can become possible. From an ethical perspective, as techniques have developed and the medical possibilities offered by gene therapy for improving health and preventing diseases have gradually materialized, its acceptance is becoming more widespread. This is exemplified by the findings of the U.S. Committee of the National Academies of Sciences, Engineering, and Medicine [36] in *Human genome editing—Science, ethics, and governance*, and the recommendations of the U.K. Nuffield Council on Bioethics [37] in *Genome editing and human reproduction: Social and ethical issues*, which considered germline editing as one possible application.

Computational techniques attempting to evaluate the effects of mutations or gene variants have been developed, although their accuracy needs to improve dramatically before they can become applicable to personalized human genetic evaluation or treatment [38]. Similarly, while extensive libraries of human SNPs have been compiled, including dbSNP, HapMap, SNPedia, and aggregating sites [39], the information is far from actionable as far as modifying multiple personalized SNPs is concerned. The ability to locate or be able to computationally estimate a complete set of the low-effect causal SNPs requires knowledge that may take decades to gain.

Gene editing technologies may also be a few decades away from the time when they can be used routinely, with the same low risk as applying an influenza vaccination, to modify a large number of gene variants distributed across the human genome. The latest gene editing technique, CRISPR-Cas9 [40], has supplemented and mostly replaced older technologies, such as zinc-finger nuclease (ZFN) [41] and transcription activator-like effector nuclease (TALEN) [42], although, for some applications, these older techniques continue to be more appropriate. While its selectivity and on-target precision have improved, CRISPR is still the most effective in gene knockdown operations. For modification and repair, only a small fraction of CRISPR operations—using homologous repair with a template or a sister chromatid sequence—succeed. A recent advance, reported by Smith et al. [43], proposed base editing with reduced DNA nicking, allowing for the simultaneous editing of >10,000 loci in human cells. CRISPR, which is only six years old, remains to be a rapidly developing technology that holds great promise. Synthetic genomics [44,45] could be another promising future technology. Synthetic genomics techniques could also help in developing the precise mapping of the effects of gene variants on disease phenotypes. If none of these approaches ultimately succeed in becoming reliable enough for the purposes of gene therapy, it is almost certain that a new, more suitable technique will be invented.

Changes in lifestyle and medical care, including the prevention and treatment of infectious diseases, have extended longevity over the last century, and this trend is projected to continue. This increased longevity is partly due to medical advances, helping people to live and function decades after first being diagnosed with historically deadly or debilitating illnesses. Preventive gene therapies may also become a future factor in prolonging health span. Actuarial science has tracked human mortality trends for centuries. The Gompertz–Makeham law of mortality, which was established more than 150 years ago, depicts an exponential increase in the rate of human mortality after the age of 30 [46,47]. While the parameters of the Gompertz–Makeham law continue to be adjusted, the principle remains valid. The apparent squaring of the mortality curve—the so-called compression of morbidity and mortality into older ages—implies that the maximum human lifespan is likely limited to about 120 years of age [48,49,50].

Within the next few decades, gene therapy techniques and genetic knowledge may sufficiently advance to support prophylactic gene therapy to prevent late-onset diseases. It may be timely to evaluate the extent of the effects that future gene therapies may have on delaying the onset of LODs or preventing them entirely.

The goal of this study is to establish how the proportional hazards model and multiplicative genetic architecture can be used to map the polygenic risk to hazard ratio of succumbing to common late-onset diseases with advancing age and apply this mapping to quantify the effects of hypothetical future prophylactic gene therapies. As its foundation, this study used earlier research [51], which reviewed epidemiology, heritability, and polygenic risk models, and developed a simulational basis for the analysis of eight of the most common diseases: AD; T2D; cerebral stroke; CA; and breast, prostate, colorectal, and lung cancers. Computer simulations in this study quantified the correlation between the aging process, the polygenic risk score (PRS), and the change in the hazard ratio with age—using as inputs the clinical incidence rate and familial heritability—and estimated the outcomes of hypothetical future prophylactic gene therapy on the lifetime risk and age of onset for these eight LODs, they also estimated the lifetime risk increase associated with longevity gains.

## 2. Results

### 2.1. Characteristics of the Aging Coefficient

**Figure 1:**
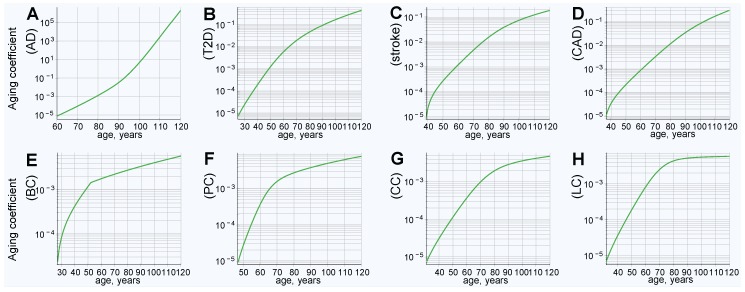
Aging coefficients reflect increases in late-onset disease (LOD) liability with age, based on the clinical incidence rate and model genetic architecture polygenic risk score (PRS). (**A**) Alzheimer’s disease (AD), (**B**) type 2 diabetes (T2D), (**C**) cerebral stroke, (**D**) coronary artery disease (CAD), (**E**) breast cancer, (**F**) prostate cancer, (**G**) colorectal cancer, and (**H**) lung cancer.

The clinical incidence rate pattern was used to map the hazard ratio from the PRS of individuals diagnosed with an LOD at each year of age for eight LODs: AD; T2D; cerebral stroke; CAD; and breast, prostate, colorectal, and lung late-onset cancers. Knowledge of the hazard ratio for any given PRS value at any given age (the aging coefficient) is what is needed for further calculations and simulations modeling gene therapy and the effects of life expectancy increases. The aging coefficients were discovered for these LODs, as described in the Methods section “Aging coefficient discovery simulation steps”, and can be seen in Figure 1.

It is interesting to note how the magnitude of the aging coefficient changes with age for the analyzed diseases. The range of values spanned by the aging coefficient and its maximum value, seen in Figure 1A, is larger for AD than for all other analyzed LODs. This can be ascribed to two factors that distinguish AD: (1) The steepest rise of incidence rate and cumulative incidence; and (2) the highest heritability of the reviewed LODs, resulting in the highest variance according to our genetic architecture model. This larger variance results in more extreme values of high and low PRSs in the population and, therefore, the age coefficient multiplier is necessarily the smallest at young onset ages; with the opposite being true at older ages. T2D, CAD, and stroke show a comparatively moderate progression and maximum projected aging coefficient values, while cancers still show a smaller maximum magnitude. Lung cancer possesses both the lowest heritability and the lowest cumulative incidence and, consequently, the values discovered by simulation for the lung cancer aging coefficient (seen in Figure 1H) almost precisely match its incidence rate. In a limit case of an LOD characterized by a PRS that remains constant with age and a very low incidence rate, the aging coefficient should be identical to the incidence rate.

### 2.2. Longer Life Expectancy Corresponds to Increasing Lifetime Risk

**Figure 2:**
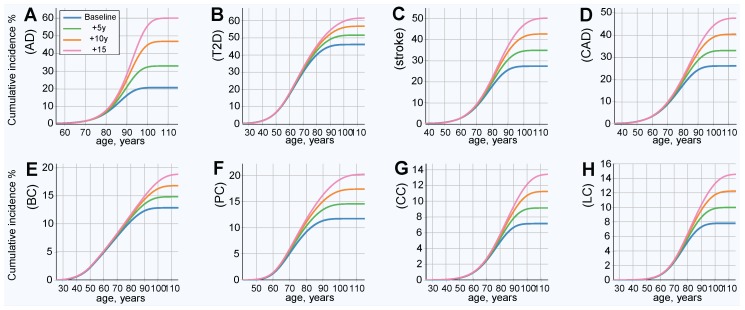
Projected LOD cumulative incidence and lifetime risk increase for life expectancy increases of 5, 10, and 15 years (baseline without gene therapy). (**A**) Alzheimer’s disease, (**B**) type 2 diabetes, (**C**) cerebral stroke, (**D**) coronary artery disease, (**E**) breast cancer, (**F**) prostate cancer, (**G**) colorectal cancer, and (**H**) lung cancer. This is the baseline scenario, without gene therapy or other health improvements for the plotted LOD. It represents the case where life expectancy increases due to causes other than the plotted LOD. Lifetime risk (lifetime cumulative incidence) corresponds to the lifetime (rightmost) values of the plots.

**Figure 3:**
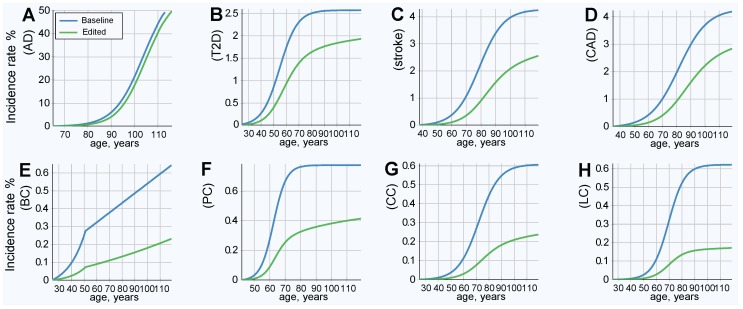
LOD incidence rate patterns, baseline and after emulated gene therapy. (**A**) Alzheimer’s disease, (**B**) type 2 diabetes, (**C**) cerebral stroke, (**D**) coronary artery disease, (**E**) breast cancer, (**F**) prostate cancer, (**G**) colorectal cancer, and (**H**) lung cancer. All individuals in the population had emulated corrective gene therapy editing of, on average, 15 single nucleotide polymorphisms (SNPs) (corresponding to an odds ratio (OR) multiplier of 0.25).

The modeled increased life expectancy in the baseline scenario, without prophylactic gene therapy, is displayed in Figure 2. This analysis corresponds to the baseline incidence rate, represented by the blue line in Figure 3. All analyzed LODs show a significant lifetime risk increase with every five years of life expectancy extension. This is most prominent with AD, the incidence of which nearly triples with an extension of life expectancy of 15 years, while the incidence rate less than doubles for the rest of the LODs. The incidence rate density (as seen in Figure S1) shows the relative incidence increasing while the peak of incidence shifts toward older ages.

### 2.3. Lifetime Risk Estimates for Discrete Hazard Ratio Multiples

Having the aging coefficient, it is easy to recalculate lifetime incidence risk for a range of hazard ratios (HRs). Population mortality is one of the principal limiting factors on the lifetime risk of an LOD, and shifting the mortality curve (and, thus, emulating longer life expectancy) reveals how incidence rate and lifetime risk would change if life expectancy were to increase.

Figure S2 shows a grid display with HR ranging from 16.0 to 0.0625 and average life expectancy varying from baseline to extended up to 15 years. The results of these calculations show that the lifetime risk is proportionate to the HR, as long as lifetime risk is relatively low (below 50%) for all reviewed LODs, even though they display quite varied incidence patterns. Lowering the HR, through gene therapy or other means, implies proportionately lowering the lifetime risk, assuming life expectancy remains constant, as summarized in Table 1. For instance, lowering the HR four-fold is accompanied by a four-fold (or 400%) drop in lifetime risk. The only exception is AD, for which the lifetime risk decreases at a slower-than-proportionate rate—a possible explanation could be the near-exponential incidence rate increase to very advanced ages for AD, while all other LODs analyzed here can be approximated by a flattened or linear progression at a more moderate old age [51]. Figure S2 shows a dramatic increase in the lifetime risk of LODs with every five extra years of life expectancy. Evidently, with increased life expectancy, the projection approaches certainty for higher HRs and increasing life expectancies. This is most prominent for AD and T2D, which can be explained by the high heritability and high later-age prevalence of these LODs, leading to a significantly higher risk that individuals will become ill earlier. Furthermore, at an advanced age, the remaining lower-risk individuals are those who constitute the majority of incidence cases [51]. As a result, the modeled high-PRS individuals show improvement in delaying the statistical disease onset, while the lifetime risk may remain almost as high.

The application of the aging coefficient and life expectancy increases makes it simple to estimate the onset delay if the PRS were changed, as in the case of prophylactic gene therapy. Table 2 and Table 3 show the values of shifts in the onset delay on the cumulative incidence slope, at 30% of the lifetime risk and for the full lifetime risk, respectively. Emulating longer life expectancy raises lifetime risk, and we see a more complex picture than a mere proportionate decline in risk, depending on the incidence and heritability patterns for each LOD. The AD lifetime risk exceeded the baseline within approximately 1–4 years of longer life expectancy. It would take approximately 10–15 years of longer life for T2D, stroke, and CAD to approach or slightly exceed their respective baseline lifetime risks. In nearly all calculated scenarios, it took significantly longer than 15 years for cancers to exceed their baseline lifetime risks.

Reviewing the first four LODs in the first row and, to a lesser extent, AD and T2D in the second row of Table 3, it is noticeable that for high PRS values (i.e., 16.0 and 4.0) the response was lower than for the final row and also lower than for the slope onset delay in Table 2. Figure S2 shows that the likelihood of becoming ill is modeled as a near certainty for these values. The simulation results of aggregating outcomes for the population in the following section provide additional generalization and confirmation of these patterns.

### 2.4. Results of Simulated Gene Therapy Lowering Population PRS

**Figure 4:**
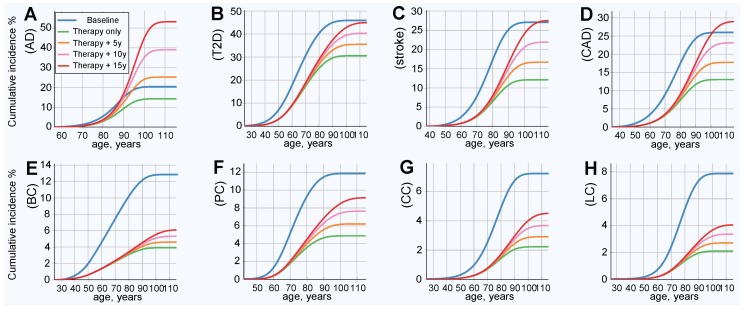
Cumulative incidence and lifetime risk following therapy reducing the population risk fourfold. (**A**) Alzheimer’s disease, (**B**) type 2 diabetes, (**C**) cerebral stroke, (**D**) coronary artery disease, (**E**) breast cancer, (**F**) prostate cancer, (**G**) colorectal cancer, and (**H**) lung cancer. All individuals in the population had emulated corrective gene therapy editing of, on average, 15 SNPs (corresponding to an OR multiplier of 0.25). Result of gene therapy and results in increases in life expectancy of 5, 10, and 15 years. Lifetime risk (lifetime cumulative incidence) corresponds to the lifetime (rightmost) values of the plots.

The results of this simulation show an improved (lower) incidence rate after treatment, as is shown in Figure 3; and a corresponding decrease in lifetime risk, as presented in Figure 4. Figure 4 also serves as a good qualitative illustration of the results of the previous section. Comparing the baseline (blue line) to therapy, with life expectancy (green line) held constant, shows a significant improvement in both the lifetime risk and age of onset for all LODs. The lifetime risk is lower for all LODs and shows a delay of approximately a decade in the incidence rate curve for T2D, stroke, and CAD. AD benefited the least, and cancers showed the most significant improvement. Table 4 illuminates the results detailed earlier from a slightly different perspective. With life expectancy unchanged, the lifetime risk decreased by 30% or more for AD and T2D, by more than 66% for colorectal and lung cancer, and by 50% or more for the remaining four LODs.

Emulating longer life expectancy resulted in increased lifetime risk. The AD lifetime risk exceeded the baseline within approximately 3 years of longer life expectancy. It took 15 years of longer life for T2D, stroke, and CAD to approach or slightly exceed their respective baseline lifetime risks. Nevertheless, even with longer life expectancies, the onset remained delayed, compared to the baseline. All cancers remained far below their baseline lifetime risks, even with a 15-year longer life expectancy. The risk remained lower than the baseline for all analyzed cancers after an increase of 15 years in life expectancy, any LOD with a relatively high heritability and low prevalence should similarly benefit. The results are summarized in Table 4, along with relevant LOD statistics.

## 3. Discussion

For the purposes of this hypothetical treatise, it was assumed that it is possible to precisely identify individual gene variants and their detrimental or beneficial effects, then, use gene therapy to modify a large number of detrimental variants. Rather than analyzing arbitrary synthetic choices of heritability and disease incidence progressions, eight LODs were chosen as a case study. Using this approach allowed us to relate the findings to some of the highly prevalent LODs that cover the broad spectrum of heritability and disease incidence patterns and—while keeping in mind that the results are a model view, with each of the reviewed LODs certainly possessing deeper specific causal mechanisms—it allowed us to make generalizations about lifetime risk changes if the LOD risks were lowered by some intervention, in this case, by gene therapy. This hypothetical gene therapy model was applied to estimate what would happen to LOD progression as the population ages. Conceptually, gene therapy here does not consider additions of artificially designed genomic sequences, but rather, only corrections made to typically low-effect heterozygous in-population gene variants, that is, a correction of a detrimental variant to a naturally occurring neutral state. For the sake of simplicity, the model used SNP distributions, though the same would apply (albeit with a higher degree of complexity) to gene therapy using other gene variant types.

This study does not evaluate potential obstacles due to pleiotropy, defined for the purposes of gene therapy as the possible negative effect on other phenotypic features of any attempt to prevent an LOD by modifying a subset of SNPs [52,53]. The high-risk individual PRS is caused by numerous variants. In this model, these are normally distributed in the population. There is a relatively small difference in the absolute number of detrimental alleles between the population average and higher-risk individuals. Arguably, for the purpose of personalized prophylactic treatment, it will be possible to select a small fraction of variants from a large set of available choices (as seen in Table 4) that do not possess antagonistic pleiotropy, or perhaps even select SNPs that are agonistically pleiotropic with regard to some of the other LODs.

Applying the modeled aging coefficient to evaluate the impact of longer life expectancy on lifetime risk confirms the long-standing observation that aging itself is the predominant risk factor for many late-onset diseases and conditions. The calculations applying the discovered aging coefficient to the discrete hazard ratio values showed a delay in onset incidence for all analyzed LODs. The lifetime risk decreased in proportion with a decrease in hazard ratio, as long as the absolute value of lifetime risk remained low. With the introduction of an emulated life expectancy increase, the lifetime risk increased. The lifetime risk increase with age was most prominent for AD. In those countries with longer life expectancy, the lifetime risk of AD is usually higher, as was demonstrated by Wu et al. [54], using the example of Japan. These results confirm, once more, that if mortality from all causes is lower (resulting in a longer life expectancy), AD is an LOD that exhibits a rapid rise in advanced-age prevalence. It would be difficult to limit the prevalence of AD, which is delayed only by approximately 3 years with the modeled level of therapy. AD may require a higher number of gene edits, likely postponing the possibility of more effective treatment to a point even further in the future; yet, any improvement would be welcome. It is possible that a pharmaceutical intervention targeting a causal metabolic pathway or immune or inflammatory response may be more effective for AD, although past announcements that generated false hope regarding breakthroughs through these kinds of approaches are too numerous to cite.

The Framingham General Cardiovascular Risk Score included age as one of the major risk factors for stroke and CAD [55]. Boehme et al. [56] showed a similar pattern for T2D, which the results of the current study were in agreement with. For T2D, stroke, and CAD, lifetime risk will regain pretreatment baselines within 10 to 15 years of longer life, which is equivalent to delaying the average onset age of these LODs by as many years. Based on heritability and incidence rate combinations, prophylactic gene therapy holds the potential to bring significant and longer-lasting benefits for cancer prevention, even with a similar or smaller number of edited gene variants than for the more prevalent diseases. The potential limitation of this study is the possibility that GWAS (and other future techniques) will have difficulties in finding a sufficient number of common low-effect SNPs to decrease the disease liability to the level simulated in this research, or that gene-environment effects will not follow Cox’s proportional hazards model [57,58] for some of the late-onset polygenic diseases. The likeliest candidate is lung cancer, which has the lowest heritability and is the most environmentally affected of all cancers reviewed here. For lung cancer, addressing the polygenic risk of smoking [59], as well as genetically influenced carcinogenicity of smoking on an individual level [60] and environmental improvements may allow for similar amelioration of disease liability. Additionally, when such advanced gene therapy technologies become available, preventing monogenic, highly detrimental variants will be simple, and the combination of therapies can bring about even more substantial improvements in both individual and population-wide health outcomes.

Gene therapy simulation scenarios analyzing population statistics showed decreases in LOD incidence and delays in LOD onset. These simulations also showed the increase in lifetime risk with emulated longer life expectancy. Such estimates may be important for evaluating population health and well-being and the potential financial impact on healthcare systems. The estimates in this study, based on the proportional hazards model and multiplicative genetic architecture using the aging coefficient, allowed for an estimation of these effects accounting for a model genetic architecture of the LODs, rather than a more simplistic calculation based primarily on the statistical shape of the incidence rate progression. In a study, aptly titled “Projections of Alzheimer’s disease in the United States and the public health impact of delaying disease onset," Brookmeyer et al. [61], it was estimated that an intervention that achieved a two-fold AD hazard ratio decrease would shift the exponential rise curve of AD by five years, leading, in the long term, to a twofold decline in the cumulative incidence and prevalence of AD when accounting for mortality. The simulation reflecting age-related change in PRS distribution demonstrated that the positive effect on the lifetime risk of AD would be significantly lower than projected by the above study, in the case of preventative gene therapy. While AD has emerged as one of the most difficult diseases to prevent, LODs with low cumulative incidence, such as cancer, exhibit enduring improvement under this model.

Even though each LOD was analyzed independently in this study, prioritizing certain LODs for preventative therapy, in practice, could have a significant effect on other conditions not specifically targeted for treatment. For example, T2D is one of the diseases that causes the most comorbidities, accelerating the onset of cardiovascular and other diseases, sometimes by decades [56]. For this reason, preventative treatment of T2D could mean improvements in health or delays in the presentation of a range of LODs, either independently of or in addition to treating their specific gene variants.

## 4. Methods

### 4.1. Conceptual Summary

This section briefly summarizes the concepts of the earlier research [51] used as a foundation for this study. The subsequent sections will describe this study’s simulation flow and implementation.

#### 4.1.1. Cox’s Proportional Hazards Model

According to Chatterjee et al. [58], the conditional age-specific incidence rate of the disease, I(t|G), defined as the probability of developing the disease at a particular age *t* given that a subject has been disease-free until that age, can be modeled using Cox’s proportional hazards model [57]:

(1) $$I\left(t|G\right)=I_{0}\left(t\right)\cdot exp\left(\sum_{k}b_{k}G_{k}\right),$$

where $G=(G_{1},\ldots ,G_{k})$ is the multiplicative effect of a set of risk factors on the baseline hazard of the disease $I_{0}\left(t\right)$. The set of age-independent variables in *G* could include genetic and environmental risk factors, as well as their interaction terms.

According to Chatterjee et al. [58], if it can be assumed that environmental risk factors are independent of the SNPs, the “post-GWAS epidemiological studies of gene-environment interactions have generally reported multiplicative joint associations between low-penetrant SNPs and environmental risk factors, with only a few exceptions”. This means that the polygenic score $G=\sum_{k}b_{k}G_{k}$, as the lifelong characteristic of each individual, is used multiplicatively with $I_{0}\left(t\right)$, which encompasses environmental and aging effects.

The simulations in this study operate on model genetic architectures of the analyzed LODs, not a complete GWAS map of their experimentally discovered SNPs, because GWAS-discovered sets can explain only a fraction of the heritability of these LODs. These model genetic architecture SNPs are treated as “true” causal for disease liability and heritability variants, as discussed in Chatterjee et al. [58], contrary to GWAS-linked SNPs—and it is assumed that they can be accurately identified for the purposes of personalized gene therapy.

#### 4.1.2. Allele Distribution Models

The allele distribution models were based on [62,63]. The allele scenarios were implemented in the simulations identically to those in the earlier research, see [51] for a comprehensive description.

The common allele low effect size genetic architecture model (Scenario A, Table 5) was expected to be most suitable for explaining the heritability of the analyzed LODs, and all results were reported using this scenario. For verification, all simulations were also performed using a more extreme rare allele medium effect size model (Scenario B, Table 5), and the results for lifetime risk were essentially identical to those in Scenario A. Any material difference in the results would have warranted additional investigation. As the results did not differ materially, separate figures are not presented for Scenario B—the corresponding simulation data are available in Supplementary Data. The number of variants needed for the Scenario A LODs is summarized in the Results section.

#### 4.1.3. LOD Incidence Rate Functional Approximation

The incidence rate functional approximations and source data are detailed in Oliynyk [51]. The yearly incidence rate logistic and exponential regressions for the functional approximation of the LODs, based on the available clinical incidence statistics, are illustrated in Figure S3. The logistic approximations were used for all LODs except breast cancer, for which the exponential followed by linear regression more accurately approximated the incidence rate pattern.

### 4.2. The Aging Coefficient: Mapping PRS to Age-Dependent Probability of LODs

Earlier research [51] showed that the incidence rates for these LODs increase as individuals age and, statistically, the PRSs of individuals diagnosed at older ages decline. Following the multiplicative model of PRS and environmental effects, from a statistical perspective, two processes are balancing disease incidence—the aging or environmental effect increases the average susceptibility of the population, and this makes it more likely that older individuals will become ill. Individuals with higher PRSs are more likely to become ill and, with higher incidence rates, a larger fraction of these individuals have already become ill with every additional year of age and are therefore excluded from the high-risk pool for the following years of age. The aging process continues, as reflected in the incidence rate rising or remaining high in subsequent years.

Statistically, individual PRSs and environmental effects including aging affect the probability of a person becoming ill [58]. According to Cox’s proportional hazards model [57], in every year of age, each individual can be assigned a hazard ratio which describes that individual’s risk of being diagnosed with an LOD. The goal is to uncover the mapping between PRSs and individual hazard ratios on a yearly basis:

(2) $$R_{u}\left(t\right)\propto A\left(t\right)\cdot G_{u},$$

where $R_{u}\left(age\right)$ is the hazard ratio of *u*th unaffected individual, and *t* is the individual’s age in years. The PRS $G_{u}$ remains constant for each individual for life, and the multiplier A(t) drives the age-related increase in LOD incidence—A(t) will also be called the “aging coefficient”.

It may be possible to map or “discover” A(t) by applying the yearly incidence rate in a population to the PRS distribution, based on an LOD genetic allele architecture, through the population simulation flow described in the next section. The discovered A(t), then applied to the population simulation on a yearly basis, should precisely reproduce the initial LOD incidence rate pattern. Later, building a population with a modified PRS to emulate the effect of gene therapy and simulating aging of this population by applying A(t), it will be possible to find the corresponding incidence rate and other resulting statistics.

In addition to the age-related change in the PRS distribution described above, the aging coefficient automatically incorporates other miscellaneous environmental effects that accumulate with age, as these are all reflected in the yearly incidence rate. The term “aging coefficient” is used throughout this publication, rather than “environmental effect”, to emphasize that it is an aggregate age-dependent parameter.

A useful parameter in the simulation and analysis is the incidence rate density D(t), which depicts LOD incidence contributions relative to the initial population count at yearly increments, adjusted for mortality:

(3) D(t)=I(t)·S(t),

where I(t) is the yearly incidence at age *t*, and S(t) is the survivor rate from the US Social Security Actuarial Life Table [64]. Integrating the area under the curve, or summing up discrete yearly values, corresponds to an LOD cumulative incidence C(t)—the limit or lifetime value C(T) equals the lifetime risk:

(4) $$C\left(t\right)=\sum_{t}^{T}D\left(t\right).$$

The comprehensive description of the simulation steps, procedures, validation, and statistical analysis is available in Supplementary Materials Chapter S1.

### 4.3. Data Sources, Programming, and Equipment

The population mortality estimates from the [64] provide annual death probability and survivor numbers, up to 119 years of age, for both men and women.

Disease incidence data were obtained from the following sources: Alzheimer’s disease [61,65,66,67], type 2 diabetes [56], coronary artery disease and cerebral stroke [68], and cancers [69,70].

The simulations were performed on an Intel Xeon Gold 6154 CPU-based 36-core computer system with 288GB of RAM. The simulation was written in C++, and the source code can be found in Supplementary Data.

The final simulation data, additional plots, R scripts, and executables are also available in Supplementary Data. Intel Parallel Studio XE was used for multithreading support and the Boost C++ library for faster statistical functions—the executable can be built and function without these two libraries, with a corresponding slowdown in execution.

## 5. Conclusions

In this study, computer simulations mapped polygenic risk to the hazard ratio of being diagnosed with eight common LODs, based on their known heritability and incidence rates, under the proportional hazards model and multiplicative genetic architecture. The resulting mapping—the aging coefficient—enabled the researcher to quantify the population effects of the emulated prophylactic gene therapy, alongside longevity increases. Computer modeling and simulations deal with simplifications and generalizations of biological processes, and aim to make predictions about the behavior of the modeled systems when modifying parameters of a model, the conclusions of this study are made in such context. The conclusions of this study are contingent on progress in molecular genetics identifying a sufficient number of true causal SNPs for a particular LOD on an individual basis, gene editing technologies becoming capable to safely provide such a level of therapy, and prophylactic gene therapies successfully passing clinical trials and obtaining the approval of governmental agencies.

The intensive gene therapy simulated here could dramatically delay the average onset of the analyzed LODs and reduce the lifetime risk of the population. The simulations highlighted that the magnitude of familial heritability and cumulative incidence patterns distinguish the outcomes for the analyzed LODs when subjected to the same PRS decrease. This outcome can be characterized by the delay in LOD onset, that is, the estimate of the number of years it would take for each LOD to regain the pretreatment baseline level.

In summary, if gene therapy, as hypothesized here, were to become possible, and if the incidence of the treated diseases followed the proportional hazards model with multiplicative genetic architecture composed of a sufficient number of common low effect gene variants, then (a) late-onset diseases with the highest familial heritability will have the highest number of variants available for editing; (b) diseases with the highest current lifetime risk, particularly those with the highest incidence rate continuing into advanced age, will be the most resistant to attempts to lower the lifetime risk and delay the age of onset at a population level; (c) diseases that are characterized by the lowest lifetime risk will show the strongest and longest-lasting response to such therapies; and (d) longer life expectancy is associated with a higher lifetime risk of these diseases, and this tendency, while delayed, will continue after the therapy.

## Figures and Tables

**Table 1:** Lifetime risk corresponding to select discrete LOD PRSs (%).

	Highly Prevalent LODs				Cancers			
	AD	T2D	Stroke	CAD	Breast	Prostate	Colorectal	Lung
**1.0**	43.3	78.7	26.8	21.1	8.27	3.94	3.33	7.12
**0.5**	31.9	39.9	13.5	10.6	4.14	1.97	1.66	3.56
**0.25**	22.0	20.0	6.75	5.30	2.07	0.984	0.832	1.78

**Table 2:** Estimate of LOD onset delay of incidence slope at 30% of lifetime risk (values in years).

	Highly Prevalent LODs				Cancers			
	AD	T2D	Stroke	CAD	Breast	Prostate	Colorectal	Lung
**16.0** → **4.0**	4	7	10	10	21	17	13	10
**4.0** → **1.0**	3	12	13	14	31	16	16	20
**1.0** → **0.25**	4	13	11	16	37	21	19	16

**Table 3:** Estimate of LOD onset delay for lifetime risk (values in years).

	Highly Prevalent LODs				Cancers			
	AD	T2D	Stroke	CAD	Breast	Prostate	Colorectal	Lung
**16.0** → **4.0**	1	0	2	3	>40	19	24	21
**4.0** → **1.0**	2	9	14	12	>40	28	34	>40
**1.0** → **0.25**	3	20	17	14	>40	29	35	>40

**Table 4:** LOD characteristics and lifetime risk in a range of life expectancy scenarios and with prophylactic gene therapy lowering population PRS fourfold.

	Highly Prevalent LODs				Cancers			
	AD	T2D	Stroke	CAD	Breast	Prostate	Colorectal	Lung
Literature and clinical data:								
Heritability	0.795	0.69	0.55	0.41	0.57	0.40	0.31	0.10
Max yearly incidence rate	>20%	2.5%	4.4%	3.6%	<0.5%	<0.8%	<0.6%	<0.6%
Genetic model SNP count	3575	2125	1175	625	1250	600	400	100
Lifetime risk, baseline + longer life:								
+5 years life expectancy	160%	112%	128%	127%	115%	123%	127%	128%
+10 years life expectancy	228%	123%	156%	155%	130%	147%	156%	156%
+15 years life expectancy	293%	134%	184%	184%	146%	172%	187%	186%
Lifetime risk, odds ratio (OR) 0.25 therapy versus baseline:								
Therapy, unchanged life expectancy	70%	67%	44%	50%	30%	41%	31%	27%
Therapy, +5 years life expectancy	124%	77%	61%	69%	36%	52%	40%	34%
Therapy, +10 years life expectancy	191%	88%	81%	89%	41%	65%	51%	43%
Therapy, +15 years life expectancy	260%	98%	101%	112%	47%	77%	62%	52%

LODs’ heritability and clinical incidence are discussed in the Methods section. The baseline is considered 100% for lifetime risk comparisons; 160% after a 5-year life expectancy extension, because AD indicates an increase in lifetime risk (LR) by 1.6 times; and 50% after gene therapy which means half the LR, compared to the baseline value. Genetic model single nucleotide polymorphism (SNP) count is the number needed for the common low-effect genetic architecture to achieve each LOD heritability.

**Table 5:** Genetic architecture scenarios with modeled allele frequencies and effect sizes.

Scenario	MAF Range	OR Range	MAF Values	Allele OR Values
A. Common low	0.073–0.499	1.05–1.15	0.073, 0.18, 0.286, 0.393, 0.5	1.05, 1.075, 1.1, 1.125, 1.15
B. Rare medium	0.0146–0.0998	1.28–2.01	0.0146, 0.036, 0.0572, 0.0785, 0.0998	1.28, 1.463, 1.645, 1.828, 2.01

To build the genetic architecture, minor allele frequencies (MAFs) and odds ratios (ORs) were chosen using 25 possible combinations of the values in the table. Following Pawitan et al. [63], the variants were assigned to individuals with frequencies proportionate to MAF $p_{k}$ for SNP *k*, producing, in accordance with the Hardy–Weinberg principle, three genotypes (AA, AB, or BB) for each SNP, with frequencies $p_{k}^{2}$, $2p_{k}\left(1-p_{k}\right)$, and ${\left(1-p_{k}\right)}^{2}$, respectively, resulting in the normal distribution of the individual risk ORs.

## Supplementary Materials

The following are available online at https://www.mdpi.com/1422-0067/20/13/3352/s1, Supplementary Document: A document **Supplementary.PDF** containing a supplementary methods chapter and figures. Supplementary Data: A zip file **SupplementaryData.ZIP** containing the simulation executable, the source code, R scripts, batch files, and simulation results.

## Abbreviations

The following abbreviations are used in this manuscript:

AD	Alzheimer’s disease
CAD	coronary artery disease
GWAS	genome-wide association study
HR	hazard ratio
LOD	late-onset disease
MAF	minor allele frequency; customarily implying the ’effect allele frequency’
OR	odds ratio
PRS	polygenic risk score
SNP	single nucleotide polymorphism; in context of this study used synonymously with the term ’allele’
T2D	type 2 diabetes
