# Supplementary material for: Quantifying the Potential for Future Gene Therapy to Lower Lifetime Risk of Polygenic Late-Onset Diseases
Source: Int J Mol Sci. 2019 Jul 8;20(13):3352. doi: 10.3390/ijms20133352 (PMC6651814; doi:10.3390/ijms20133352)
Supplement: Supplementary file 1 [file ijms-20-03352-s001.pdf]

Quantifying the Potential for Future Gene Therapy to Lower Lifetime Risk of Polygenic Late-onset Diseases

Roman Teo Oliynyk[1,2,*]

[1]Centre for Computational Evolution, University of Auckland, Auckland 1010, New Zealand
[2]Department of Computer Science, University of Auckland, Auckland 1010, New Zealand
[*]roli573@aucklanduni.ac.nz

SUPPLEMENTARY FIGURES

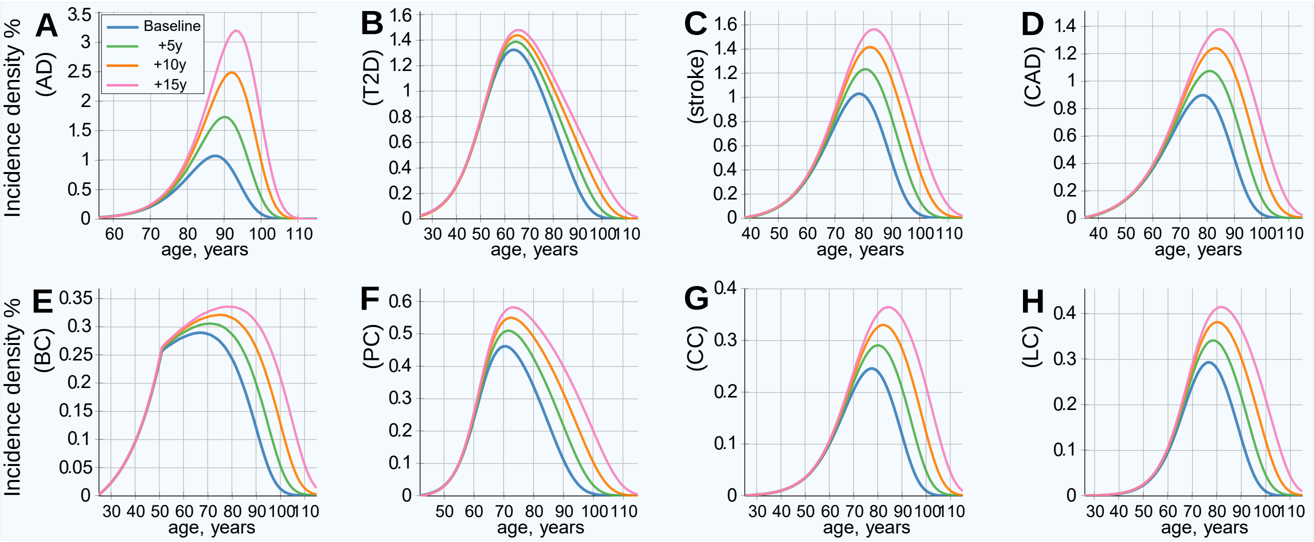

Figure S1. Baseline (without gene therapy) population cumulative incidence rate density.
(A) Alzheimer's disease, **(B)** type 2 diabetes, **(C)** cerebral stroke, **(D)** coronary artery disease, **(E)** breast cancer, **(F)** prostate cancer, **(G)** colorectal cancer, **(H)** lung cancer.
The area under the curve is equal to the lifetime risk for each scenario, accounting for mortality. Projected LOD incidence rate relative to the number of individuals at birth, baseline, and scenarios with life expectancy increased by 5, 10, and 15 years. This is the baseline scenario without gene therapy or other health improvements for the plotted LOD. This represents the case where life expectancy increases due to causes other than the plotted LOD.

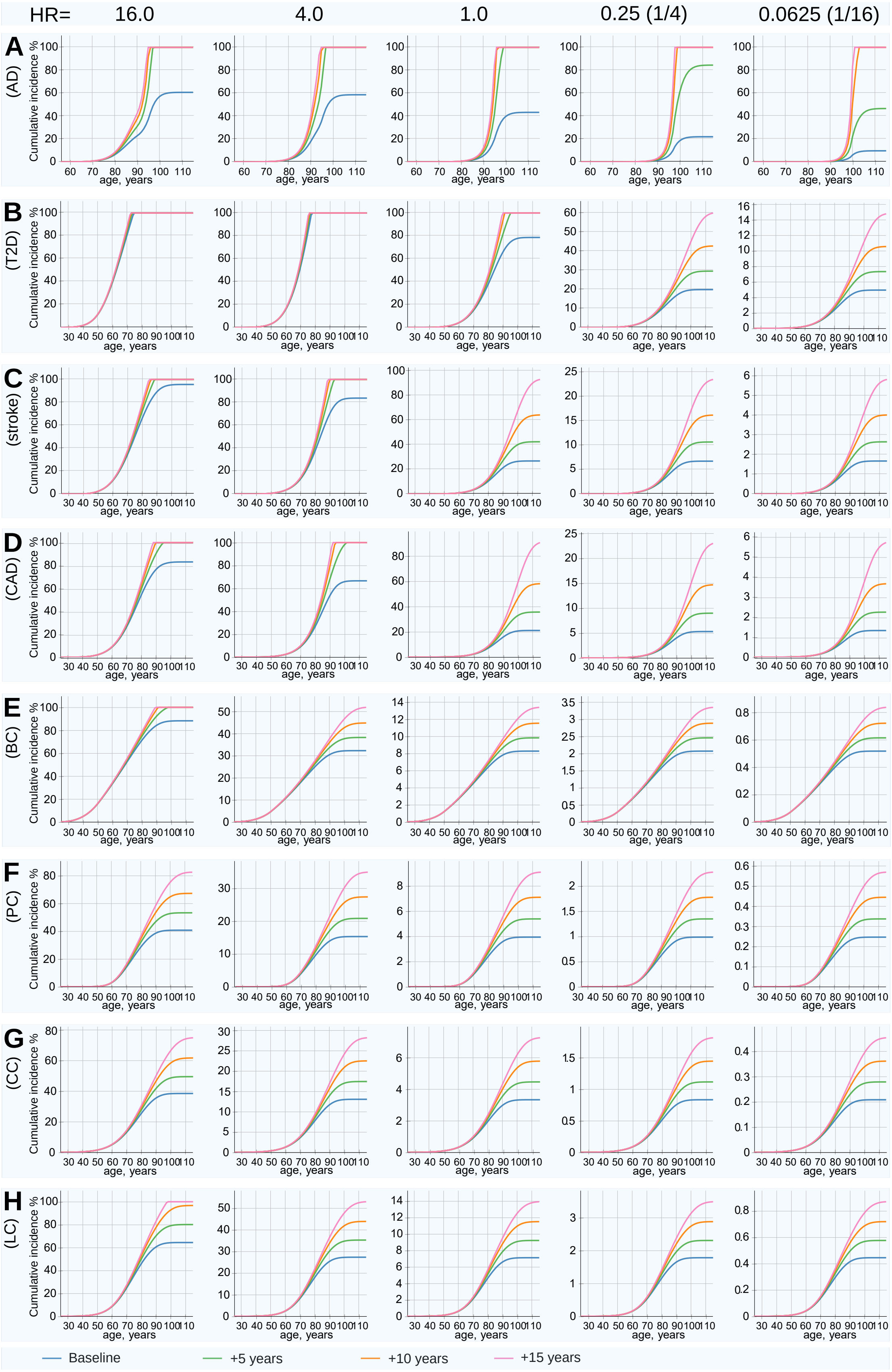

Figure S2. Matrix display of cumulative incidence and lifetime risk for discrete hazard ratio values.
(A) Alzheimer's disease, **(B)** type 2 diabetes, **(C)** cerebral stroke, **(D)** coronary artery disease, **(E)** breast cancer, **(F)** prostate cancer, **(G)** colorectal cancer, **(H)** lung cancer.
This figure shows the matrix of calculated cumulative incidence for discrete hazard ratio (HR) values relative to the population mean, and cumulative incidence increase in 5-year life expectancy increments. Lifetime risk (lifetime cumulative incidence) corresponds to the lifetime (rightmost) values of the plots. For lower HRs, the lifetime values equal to the lifetime risk are almost precisely proportionate to the HR multiple (note the scale change), while the age progression curve for each HR value shifts toward older ages. This shift is most prominent for the highest incidence LODs; the change in scale moving from higher to lower HRs visually masks the shift appearance.

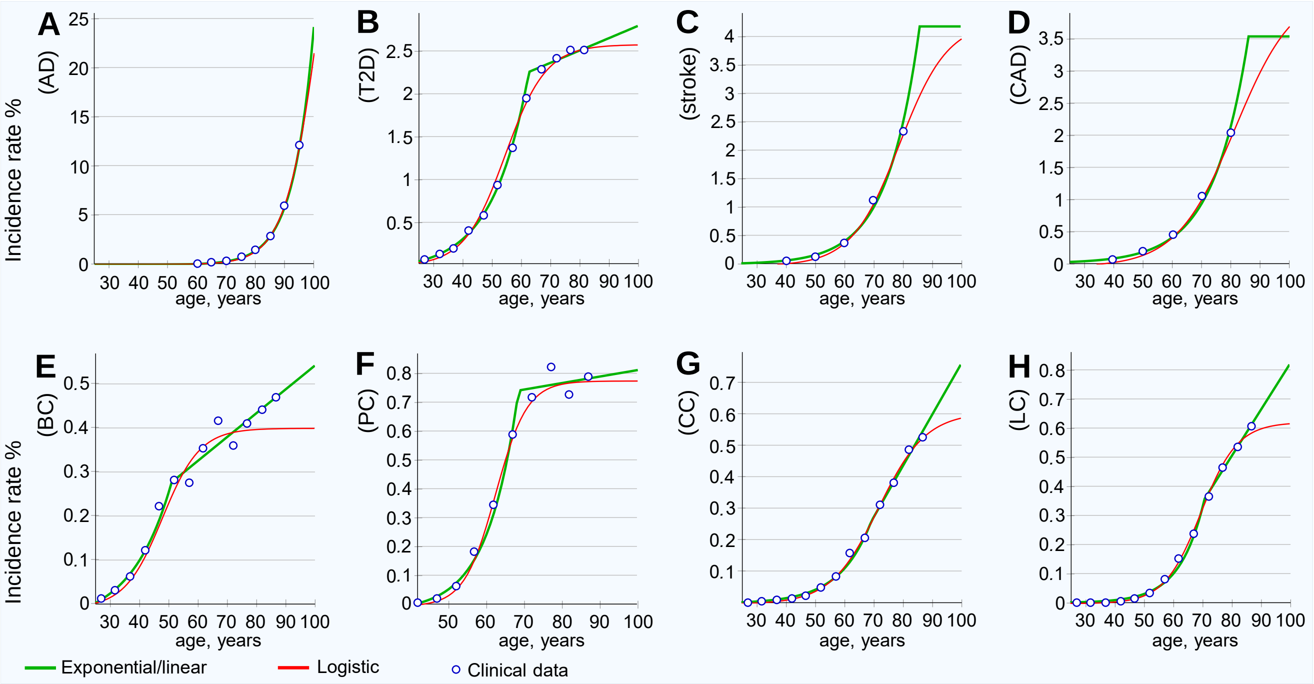

Figure S3. LOD clinical incidence rates and functional approximations.

(A) Alzheimer's disease, **(B)** type 2 diabetes, **(C)** cerebral stroke, **(D)** coronary artery disease, **(E)** breast cancer, **(F)** prostate cancer, **(G)** colorectal cancer, **(H)** lung cancer.

Two functional approximations of clinical data: exponential followed by linear and logistic.

Chapter S1: The aging coefficient simulations

Simulation steps in discovering LOD aging coefficients

1. The simulation initialization steps are performed, including allocating population objects, assigning individual PRSs based on the modeled genetic architecture, and building an incidence rate functional approximation.
2. The simulation works as an iterative procedure, where the values of $A(t)$ are matched at advancing ages, starting at the age at which the incidence rate first becomes noticeable for an LOD, denoted as $t = T_0$. The initial value is relatively immaterial, and each simulation begins with the value $A(T_0) = 0.001$, The iterative process, by following steps 3 and 4, rapidly finds a close match value. For instance, in the case of AD, age $T_0 = 39$ years, $I(39) = 2.06 \cdot 10^{-6}$, and the resulting $A(39) = 5.85 \cdot 10^{-9}$. $A(t)$ discovery for the next year commences with the value for the previous year and as a result requires slightly fewer iterations to find the match.
3. As the $A(t)$ value is applied to all individuals' PRSs, a first estimate of each individual hazard ratio for this year is produced. Based on the resulting probabilities, a number of individuals will be diagnosed at age t. This number is simply $N_d(t) = I(t) \cdot N_u(t)$, where $N_u(t)$ is the remaining healthy population and $N_d(t)$ is the number of individuals expected to be diagnosed.
4. The simulation verifies how well the result matches the expected incidence rate for the LOD at this age, and a better-matching approximation value is recalculated and reapplied to step 3. This process will iterate steps 3 and 4 until a predetermined level of accuracy is achieved, with the aim of attaining 0.1% reproduction accuracy for ages with significant incidence rates.
5. This cycle of steps 3 and 4 is then rerun a predetermined number of times (with 10 repetitions being the default), with iterations alternatively commencing with a value $A(t)$ 10% above and below the previously determined value to account for a potential determination bias. The results of these reruns are averaged, and the resulting variance is evaluated and recorded.
6. The validated aging coefficient $A(t)$ from step 5 is then applied to the step 3 operation one final time in order to sample the individuals diagnosed at this age out of the population. Statistically, the highest-PRS individuals are likelier to be diagnosed, and these individuals are excluded from the healthy population.
7. The age is advanced by one year, with a now-smaller population $N_u(t+1) = N_u(t) - N_d(t)$. The simulation repeats steps 3–6 until all ages are covered—in our case, until the age of 120.

At completion, the aging coefficients that map the modeled PRS value to the individual hazard ratio for each year of age have been discovered.

Calculating cumulative incidence and lifetime risk for discrete LOD hazard ratios

The cumulative incidence is determined by applying the aging coefficients found in the discovery simulation above to a range of hazard ratio (HR) multiples relative to the population mean. As reported in the Results section, these calculations allow for the evaluation of how cumulative incidence and lifetime risk correspond to hazard ratios; this is equivalent to learning what would happen if the individual hazard ratio were changed using gene therapy (or, in principle, any intervention of a similar effect). An R script (available in Supplementary Data) calculates the incidence for a fixed value of hazard ratio G_k through the range of ages from 0 to 120:

$$I(t) = A(t) \cdot G_k,$$

and Equation (4) in the main article gives the age progression of lifetime risk. The HR values used were: 16.0, 4.0, 1.0, 0.25 and 0.0625; the script could be modified for any set of desired values.

Here, an R script takes the aging coefficient as the input and calculates the lifetime risks for PRS = 1.0 (population mean risk), PRS = 0.5, and PRS = 0.25 and the numbers of years by which life expectancy must increase in order for the lower PRS value to again exceed the mean population lifetime risk, reported in the Results section for fourfold PRS changes: 16.0 → 4.0, 4.0 → 1.0, and 1.0 → 0.25.

Simulating outcomes of gene therapy lowering population polygenic risks

In this simulation, the aging coefficient discovered through the earlier simulation is applied while the population ages, and the resulting population incidence rate and lifetime risk patterns are analyzed.

It is possible to conceive any number of scenarios of prophylactic gene therapy. In the most extreme scenario, one could make an equivalent of thousands of edits for AD, and hundreds for most of the rest of the analyzed LODs, reversing all variants constituting individual PRSs to a neutral state; this would show a resulting disease risk and incidence of zero. It is likely that any models featuring so drastic an intervention would be unrealistic, and many less extreme scenarios are possible.

The simulation scenario where every population's individual PRS is decreased fourfold (OR=0.25) was chosen as a sufficient number of edits to achieve a substantial effect: It delays the lifetime risk of T2D, stroke and CAD by almost precisely 15 years and provides even better outcomes for all analyzed cancers, while highlighting the difficulty of treating

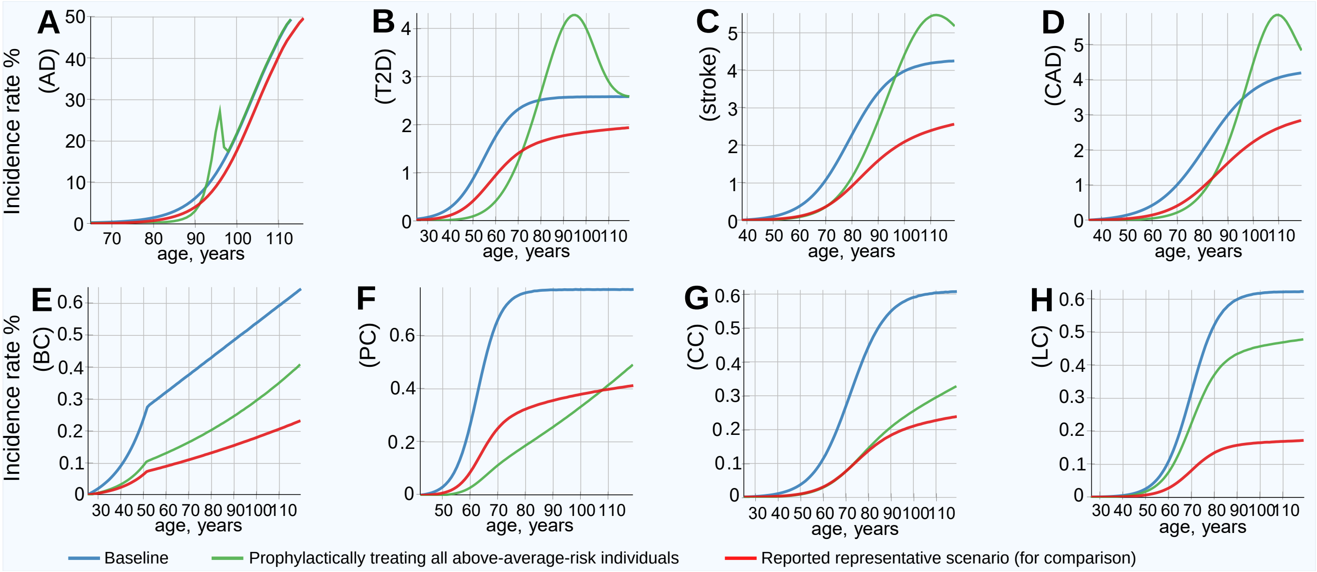

Figure S4. **Incidence rate pattern, baseline and after gene therapy. Prophylactically treating above-average-risk individuals to match the polygenic risk average of the baseline population.**
(A) Alzheimer's disease, **(B)** type 2 diabetes, **(C)** cerebral stroke, **(D)** coronary artery disease, **(E)** breast cancer, **(F)** prostate cancer, **(G)** colorectal cancer, **(H)** lung cancer.
There is a pronounced delay in early incidence for all LODs. As a result of half of the population having PRSs equal to what was previously the population mean PRS, statistically, a large number of individuals are likely to become sick at relatively similar advanced age. For the four non-cancer LODs, there is a spike in the incidence rate at a very old age, which exceeds the baseline incidence rate. The spike appears steep; the proportion of the population that remains alive at this old age is diminishing. LODs with lower incidence and heritability do not display a spike exceeding the baseline incidence. The values reported in the article's representative scenario are shown for comparison.

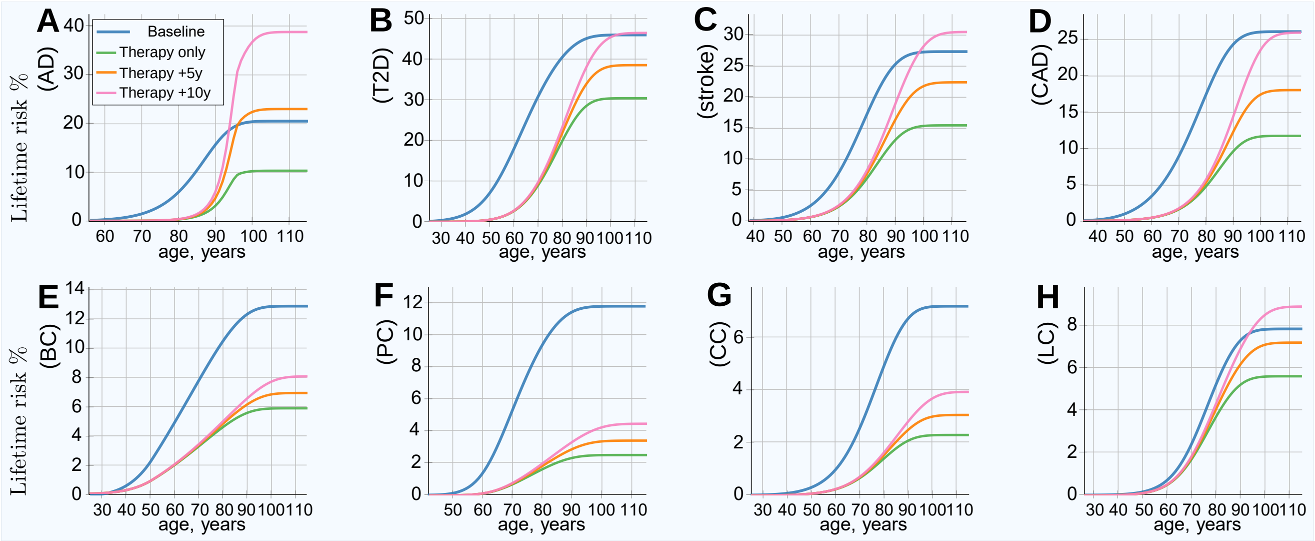

Figure S5. **Lifetime risk, baseline and after gene therapy. Prophylactically treating above-average-risk individuals to match the polygenic risk average of the baseline population.**
(A) Alzheimer's disease, **(B)** type 2 diabetes, **(C)** cerebral stroke, **(D)** coronary artery disease, **(E)** breast cancer, **(F)** prostate cancer, **(G)** colorectal cancer, **(H)** lung cancer. Showing lifetime risk after therapy, and the trends with life expectancy increased by 5 and 10 years.
The lifetime risk results are qualitatively similar to the reported scenario, though not as large, and a life expectancy increase of 10 years results in the risk exceeding the baseline for the four non-cancer LODs and lung cancer. Lung cancer showed the least improvement. This is due to the disease having the lowest reported heritability, which results in the lowest PRS variance and the fewest edits for the treated population under this scenario.

AD. A life extension of 15 years would result in an average life expectancy of about 95 years, and this may be considered as challenging the unknown limits of the squaring of the mortality curve sufficiently far for this study.
It appears most practical for a person to be born with all cells already treated, because all developmental stages are affected by the genome, which implies germline therapy and likely heritability of therapy. Nevertheless, it is not yet known what technological possibilities and ethical considerations the future will bring; therefore, without discussing the specifics of any method, it is assumed that, at birth (or age zero), the person's genome appears with the required modification. The therapy emulation is a simple arithmetic operation of reducing the individual PRS by a desired

value. Simulating a fourfold reduction in all population individuals' PRS (OR = 0.25, equivalent to β = log(OR) = -1.39) requires an edit of 15 SNPs in a common allele low-effect-size scenario with an average OR of 1.1; it is simply calculated as: $1.1^{15} \approx 4$. Choosing to edit only the largest-effect-size SNPs available in the genetic architecture, with OR = 1.15, would require approximately 10 edits: $1.15^{10} \approx 4$. The simulations were then performed with the previously discovered values of $A(t)$ and the population with the modified individual PRSs.
The lower- and higher- intensity scenarios were also simulated and were qualitatively similar to the simulation above, with a corresponding decrease or increase in lifetime risk and onset delay patterns; the above scenario was found to be the most representative and illustrative and is reported in the Results section. A somewhat different scenario, in which all individuals with elevated PRSs were treated to adjust their PRSs to that of the population mean, merited an illustration in Figure S4 and Figure S5; the lifetime risk outcomes are also qualitatively similar to those in the above simulation.

Emulating life expectancy increases

Life expectancy increases are emulated by adjusting the mortality from all causes by the desired number of years. All mortality rates from the US Social Security Actuarial Life Table [2] are shifted by 5, 10, and 15 years. This approach is supported by Zuo et al. [3], who showed that the front slope between the 25th and 90th percentiles of old-age deaths advanced with a nearly constant long-term shape as longevity increased over the past five decades. It may be not prudent to heed the opinion expressed in Zuo et al. [3] that there is "no support for an impending limit to human lifespan" indefinitely, but it may be sufficient for this study's estimates of up to 15 years of increased life expectancy. Although the Results section reports values with greater life expectancy increases, these have primarily comparative value, and > 40 years will denote even greater extensions.

The aging coefficient values and simulation accuracy

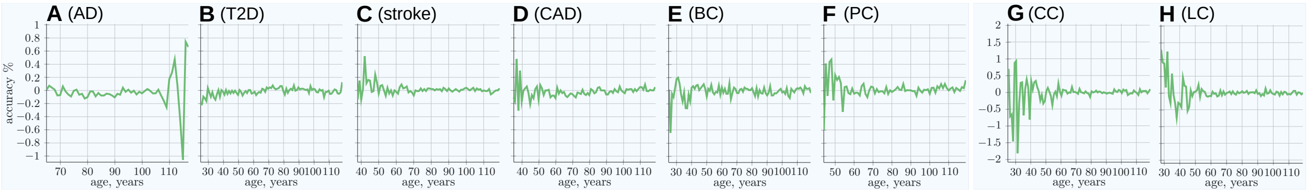

Figure S6. Accuracy of simulation aging coefficient discovery in reproducing the input incidence rate approximation.
(A) Alzheimer's disease, **(B)** type 2 diabetes, **(C)** cerebral stroke, **(D)** coronary artery disease, **(E)** breast cancer, **(F)** prostate cancer, **(G)** colorectal cancer, **(H)** lung cancer.

Figure S6 shows the resulting accuracy produced by applying the aging coefficient to eight LODs, compared to the input incidence rate approximation. The error rate begins near 2% for colorectal and lung cancers, is noticeably better than 1% for the remaining LODs, and stays below 0.2% for most of the LODs' onset range. This validation illustrates that the combined discovery and analysis reproduce the input LOD incidence rate with a high degree of precision.
The core of the simulation is the iterative discovery and application of the aging coefficient (see Equation (2) in the main article Methods), mapping individual PRSs to the hazard ratio specific to each LOD on a yearly basis, depicted in Figure 1 in the main article. The aging coefficient incorporates combined aging and environmental effects, and the rising pattern indicates the increasing magnitude of these effects with age. Figure S7 shows the PRS distribution for diagnosed individuals by age when the simulation is rerun using the discovered aging coefficient without gene editing (a baseline validation). This distribution matches the results of Oliynyk [1], in which the simulation used a direct probabilistic algorithm. A more precise indication of the accuracy of the discovered aging coefficient is shown in Figure S6, where the simulation aging reproduces the population incidence rate Equation (2) in the main article Methods.
The values with lower accuracy occur at very early and very late ages of onset, affecting a minute fraction of the total population. This in itself is the cause of the variability. As seen in Figure S1, the baseline case, and in Figure S8, the gene therapy case, the incidence rate density is very low at younger ages, when it is below $1 \cdot 10^{-5}$. Due to population mortality, even though the incidence rate is high at old ages, the number of individuals still alive after the age of 105 is small, even with an increase in the modeled life expectancy of 15 years. Only in the case of AD, the incidence of which grows exponentially to a very late age, does the remaining unaffected population become small near 120 years of age, causing the error rate to increase to close to 1%, a minuscule deviation in light of the particularly low incidence rate density at this age.
This precision is due to the use of large population sets: 25 million simulated individuals, with aggregation of 250 simulation loops (configurable), for each simulation iteration in the discovery stage and a population of one billion for the gene therapy simulation. While the analysis could certainly have been performed with smaller simulation sets,

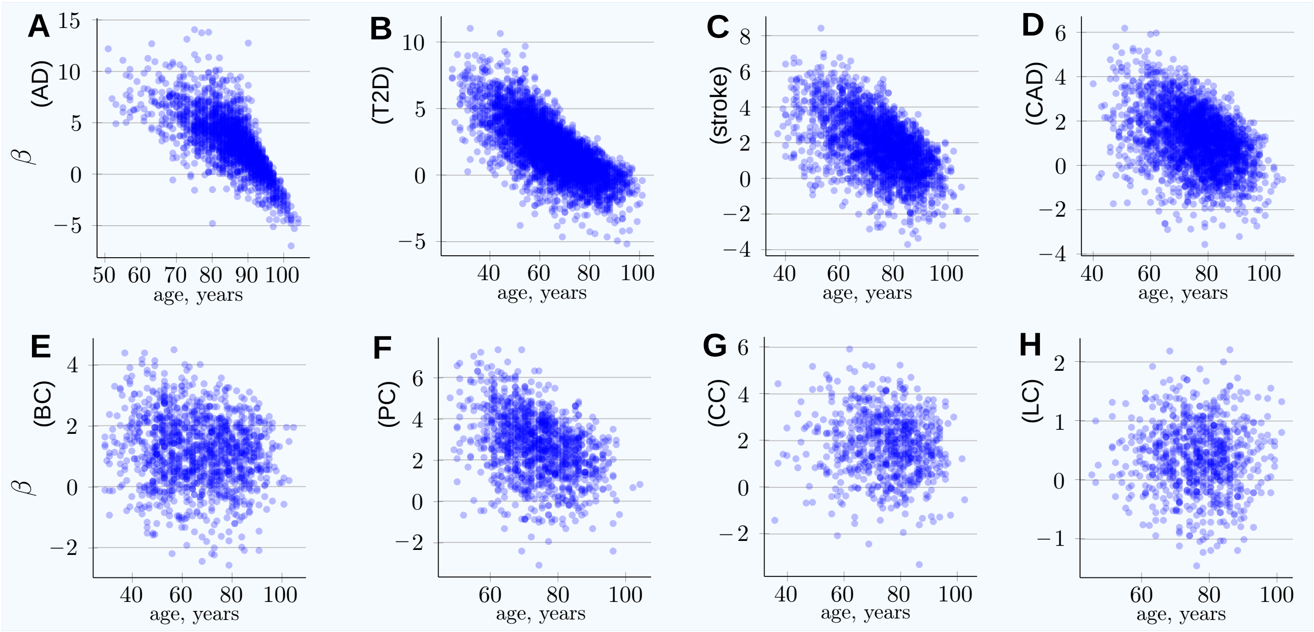

Figure S7. Aging coefficients applied to reproduce the LOD incidence distribution by age based on individual PRS.
(A) Alzheimer's disease, **(B)** type 2 diabetes, **(C)** cerebral stroke, **(D)** coronary artery disease, **(E)** breast cancer, **(F)** prostate cancer, **(G)** colorectal cancer, **(H)** lung cancer.
Polygenic scores of individuals diagnosed with an LOD as a function of age. Scatter plots show the distributions of polygenic scores for cases diagnosed as age progresses. $PRS\ \beta = log(OddsRatio)$.

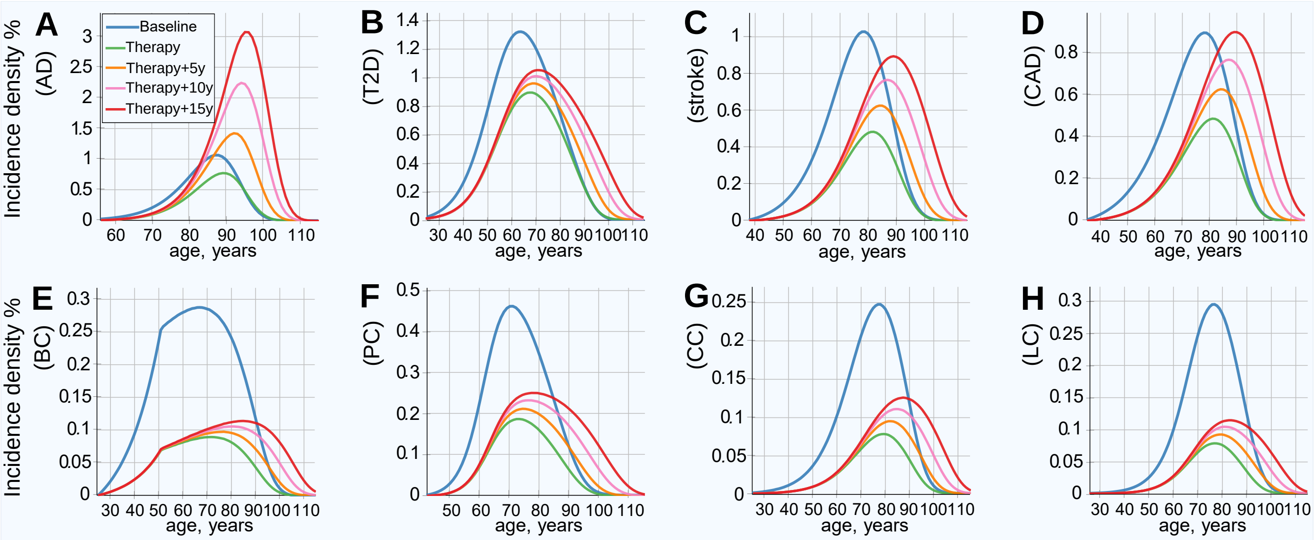

Figure S8. Population cumulative incidence rate density after emulated gene therapy.
(A) Alzheimer's disease, **(B)** type 2 diabetes, **(C)** cerebral stroke, **(D)** coronary artery disease, **(E)** breast cancer, **(F)** prostate cancer, **(G)** colorectal cancer, **(H)** lung cancer.
All individuals in the population had emulated corrective gene therapy editing, on average, 15 SNPs (corresponding to an OR multiplier of 0.25). The area under the curve is equal to the lifetime risk for each scenario, accounting for mortality. Projected LOD incidence rate relative to the number of individuals at birth, after gene therapy, and scenarios with life expectancy increased by 5, 10, and 15 years.

leveraging the available computing equipment allowed for the achievement of low statistical variance while simplifying analysis. This precision rendered the use of error bars in the graphical displays impractical.

References

[1] R. T. Oliynyk. Age-related late-onset disease heritability patterns and implications for genome-wide association studies. *PeerJ*, 7:e7168, June 2019. ISSN 2167-8359. doi: 10.7717/peerj.7168.

[2] US Social Security Actuarial Life Table. *Social Security Administration (US). Available at https://www.ssa.gov/oact/STATS/table4c6.html (accessed June 2, 2019)*, 2014.

[3] W. Zuo, S. Jiang, Z. Guo, M. W. Feldman, and S. Tuljapurkar. Advancing front of old-age human survival. *Proceedings of the National Academy of Sciences*, 115(44):11209–11214, 2018. ISSN 0027-8424. doi: 10.1073/pnas.1812337115. URL http://www.pnas.org/content/115/44/11209.
